# Empirical Blaschke Mode decomposition: Algorithm and application

**DOI:** 10.1371/journal.pone.0346738

**Published:** 2026-05-06

**Authors:** Sainan Li, Jing Wu

**Affiliations:** Department of stomatology, People’s Hospital of Longhua, Shenzhen, People's Republic of China; Michigan State University, UNITED STATES OF AMERICA

## Abstract

Periodic pulse components are common in complex signals across fields like biomedical and aerospace engineering, and their accurate extraction is crucial for monitoring. We propose a novel method, Empirical Blaschke Mode Decomposition (EBMD), which uses the Blaschke Transform to capture quasi-periodic features and a unimodal pre-segmentation strategy to define relevant spectrum bands. A group sparse filter bank decomposes the signal into fundamental modes, extracting periodic pulse features. To prevent over-decomposition, a periodic frequency similarity fusion strategy consolidates the modes into eigenmode functions. EBMD is validated through simulations and applications in mechanical vibration feature extraction, EEG denoising, and ECG signal separation, demonstrating its ability to separate signals, extract features, and reduce noise.

## 1. Introduction

Periodic pulse components appear in various signals such as ECG, EEG, EMG, mechanical vibrations, and current signals, making their analysis essential for health monitoring. For example, changes in the QRS complex of ECG signals can indicate arrhythmias and heart disease [1], epileptiform spikes in EEG signals are crucial for diagnosing epilepsy [2], motor unit action potentials in EMG signals reflect muscle activation [3], pulse characteristics in PPG signals indicate vascular elasticity [4], and vibration patterns in rotating machinery provide insights into equipment health [5]. To extract or separate these features effectively, researchers are continually developing advanced signal decomposition techniques.

Singular value decomposition (SVD) [6] is a signal denoising technique based on matrix reconstruction, involving three steps: constructing a Hankel matrix, orthogonal space decomposition, and singular value truncation. By forming a time-delay embedding matrix, SVD uses the singular value spectrum to characterize signal energy distribution and suppress noise by truncating minor singular values. While this method effectively quantifies abrupt signal changes, its key limitation lies in the lack of mathematical criteria for determining the effective rank, which can result in the loss of important components or residual noise. Singular Spectrum Analysis (SSA) [7], an extension of SVD, optimizes component reconstruction by assuming periodicity in time series. However, its performance is highly sensitive to the embedding dimension: improper dimension settings can lead to modal overlap, particularly in high-noise environments, where spectral overlap between high-frequency noise and the target signal hinders effective decomposition.

Empirical Mode Decomposition (EMD) [8] adaptively decomposes a signal into intrinsic mode functions (IMFs), making it well-suited for nonlinear and non-stationary signal analysis. However, EMD lacks a solid mathematical foundation and is sensitive to noise, leading to modal confusion and envelope issues. To address these, methods like Ensemble Empirical Mode Decomposition (EEMD) [9] and Complementary Ensemble Empirical Mode Decomposition (CEEMD) [10] introduce white noise, improving decomposition but still suffering from noise residuals and mode inconsistencies. A novel improvement, Complementary Ensemble Empirical Mode Decomposition with Adaptive Noise (CEEMDAN) [11], uses a stepwise noise addition approach, injecting adaptive white noise into the residual signal at each iteration to prevent cumulative noise effects on low-frequency components. Compared to EEMD and CEEMD, CEEMDAN reduces redundant modes, ensures decomposition completeness, and achieves lower reconstruction error.

Variational Mode Decomposition (VMD) [12] redefines signal decomposition by framing IMF extraction as a frequency-domain optimization problem. By pre-setting the number of modes and a penalty factor, VMD constructs an adaptive Wiener filter bank, achieving superior mode separation compared to EMD. However, VMD requires manual selection of parameters such as the number of modes and initial center frequencies, with no standard for evaluating decomposition quality. Additionally, the components derived from VMD are not orthogonal.

Empirical Fourier Decomposition (EFD) [13], based on spectral segmentation theory, combines enhanced spectral segmentation with zero-phase rectangular filters. While it effectively decomposes complex signals by pre-setting the number of modes, it relies on minimum value techniques for segmentation, which can lead to over- or under-segmentation. Furthermore, EFD’s use of zero-phase filters prevents effective noise removal within the segmented frequency bands.

In recent years, researchers have proposed transforming complex signal analysis into the analysis of Blaschke products. The Blaschke product is a rational function with infinite differentiability, theoretically capable of approximating complex signals with extremely high precision. Unlike traditional basis functions, the Blaschke product contains control parameters that can be adaptively adjusted based on the characteristics of the input signal, thereby flexibly matching the signal’s nonlinear and non-stationary behavior in terms of waveform, instantaneous frequency, and local instantaneous oscillatory characteristics. Therefore, the Blaschke basis is naturally suitable for processing signals with modulated components, frequency variations, or nonlinear dynamic features.

Adaptive Fourier Decomposition (AFD) [14] is a classic Blaschke product-based signal processing method. AFD essentially determines the control parameters based on the principle of maximum selection, constructing the optimal Blaschke basis that is highly consistent with the signal’s characteristics. Compared to traditional methods such as Fourier transforms and wavelet transforms, AFD has unique advantages, including high resolution, strong adaptability, and no cumulative reconstruction error. It is particularly suitable for signal analysis in complex modulation, rapid instantaneous frequency variation, and low signal-to-noise ratio scenarios. Due to the significant theoretical potential of AFD, researchers have proposed a series of improvements, such as POAFD [15], Cyclic AFD [16], BTWSV [17], BMD [18] and BLM [19]. However, AFD and its improved methods do not consider periodic pulse characteristics, making it difficult to extract internal features from signals. Periodic pulse characteristics represent the most regular and interpretable components within a signal, effectively revealing its underlying mechanisms. A key feature of periodic pulses is group sparsity frequency Structure. By leveraging this group sparse structure, we can efficiently extract intrinsic information from the signal.

Based on the analysis above, this paper introduces a novel signal decomposition method, Empirical Group Sparse Mode Decomposition (EBMD). First, EBMD applies Blaschke Transform to extract the quasi-periodic features of the original signal and implements a unimodal pre-segmentation strategy to define the spectral band range for the decomposition modes, ensuring the physical relevance of the pre-decomposed modes. Next, EBMD employs a group sparse filter bank to decompose the signal into a series of fundamental modes, effectively isolating the hidden periodic pulse components within the signal. Finally, EBMD introduces a periodic frequency similarity fusion strategy to combine the fundamental mode components into a series of eigenmode functions, addressing the issue of over-decomposition.

The contributions of this paper are as follows:

(1) EBMD employs Blaschke Transform to extract the quasi-periodic characteristics of original signal and constructs a unimodal pre-segmentation strategy, ensuring the decomposed components are interpretable.
(2) EBMD designs a novel group sparse filter bank that can precisely extract the periodic pulse features hidden within the signal and remove internal noise interference, ensuring that the decomposition result contains intrinsic information.
(3) EBMD propose periodic pulse fusion strategy to fuse the fundamental mode components into a series of IMFs, effectively overcoming the over-decomposition issue.

As a new signal decomposition method, EBMD can accurately extract the periodic pulse features hidden in the signal, remove internal noise interference, and achieve signal separation, making it of practical application value. The structure of the rest of the paper is as follows: Section II explains the EBMD theory, Section III presents experimental case analyses, and Section IV is conclusion.

## 2. Empirical Blaschke mode decomposition

### 2.1. Blaschke transform

Blaschke transform [18] uses a specific Blaschke product to perform the Szegő projection, adaptively representing complex signals as a linear combination of orthogonal Blaschke mono-components with quasi-periodic characteristics in Hardy space. This transform uncovers the hidden intrinsic features of complex signals.

In Blaschke Transform theory, any signal or system can be expressed as a linear combination of Szegő projections of Blaschke products. For a given analytic signal, the following identity holds:


$$f\left(z\right)=\sum_{i=1}^{k}\langle f_{i},e_{a_{i}}\rangle B_{i}\left(z\right)+f_{k+1}\left(z\right)\prod_{i=1}^{k}\frac{z-a_{i}}{1-{\overset{―}{a}}_{i}z}$$ (1)

where,


$$f_{k+1}\left(z\right)=\frac{f_{k}\left(z\right)-\langle f_{k},e_{a_{k}}\rangle e_{a_{k}}\left(z\right)}{\frac{z-a_{k}}{1-{\overset{―}{a}}_{k}}}$$

$\langle f_{i},e_{a_{i}}\rangle$ is the inner product of function $f_{\text{1}}$ and unitized Szegő kernel $e_{a_{i}}$, $a_{i}\in D,D=\{z\in \mathbb{C}:\left|z\right|<1\}$, f(z) is the complex signal, $B_{i}$ is Blaschke product, k is the order of Blaschke transform.

Through iterative selection the parameter $\left\{a_{\text{1}},a_{\text{2}},...,a_{n}\right\}$, Blaschke transform can adaptively map an analytic signal f(z) to the optimal power series space. The Blaschke product, also known as the Blaschke mono-component, serves as the unitized Szegő kernel for our definition of the unitary Szegő projection. The expressions for Blaschke mono-components of different orders are presented in Eq (2).


$$\left\{ \begin{array}{c} @l@B_{n}\left(z\right)=1\;n=1\\ B_{n}\left(z\right)=\frac{\sqrt{1-{\left|a_{n}\right|}^{\text{2}}z}}{1-{\overset{―}{a}}_{n}z}\prod_{k=2}^{n-1}\frac{z-a_{k}}{1-{\overset{―}{a}}_{k}z}\;n\neq 1 \end{array}\right.$$ (2)

where, n is the order of Blaschke mono-component.

Blaschke transform involves stepwise determination of the optimal parameter projection sequence $\left\{a_{\text{1}},a_{\text{2}},...,a_{n}\right\}$.

We have developed a linear density dictionary $D_{a}$ within Hilbert space and introduced the concept of quasi-periodic components. These components share similarities with periodic signals but lack a fixed period. Their pronounced periodic variations allow them to capture the inherent features of the observed system. Thus, representing complex signals as a linear combination of quasi-periodic components is a sound approach. To optimize the parameters within the dictionary for signal representation, we reformulate the Blaschke transform as an optimization problem, utilizing the autocorrelation properties of quasi-periodic components, as shown in Eq (3).


$$a=argmax\left\{\left|\sum_{m=1}^{N}R_{B_{i}}\left(m\right)/N\right|:a_{i}\in D_{a}\right\}$$ (3)

where, $B_{i}$ is the corresponding Blaschke mono-component of $a_{i}$, R is the autocorrelation function, m is the time delay, N is the number of sample points.

Through the maximization of Eq (3) in each order of the Blaschke transform, we ensure that the Blaschke mono-components that correspond to the parameters $\left\{a_{\text{1}},a_{\text{2}},...,a_{n}\right\}$ possess the most strong quasi-periodic characteristics. Additionally, for a more intuitive representation of the energy distribution among Blaschke components of different orders, we calculate the average energy intensity of these components according to Eq (4). Building upon this, we define a novel spectral representation known as the Blaschke spectrum.


$$BS(k)=\frac{\sum_{i=1}^{N}(B_{k}(i){)}^{\text{2}}}{N}$$ (4)

The Blaschke spectrum reveals the energy distribution of Blaschke mono-components across different orders and effectively captures the hidden quasi-periodic characteristics of complex signals, providing an intuitive measure of their strength. By representing complex signals as linear combinations of Blaschke mono-components, the Blaschke transform uncovers these concealed characteristics. Additionally, the Blaschke spectrum offers a novel perspective for understanding signal properties, enabling deeper insight into the intrinsic features of complex signals.

### 2.2. Unimodal pre-segmentation strategy

The core idea of the unimodal pre-segmentation strategy is to divide the spectrum into continuous intervals that satisfy the characteristics of unimodal, using statistical hypothesis testing methods. This process separates the signal into a series of mono-components.

First, the definition of a unimodal is provided: when a frequency band [a,b] shows a monotonically increasing characteristic in the segment [a,c] and a monotonically decreasing characteristic in the segment [c,b], the frequency band is considered to have a unimodal property. At this point, the spectral energy is concentrated at the peak point c, which can be statistically regarded as approximately a unimodal distribution. A signal with a spectrum that has a unimodal distribution is defined as a mono-component.

Let x(t) be the original signal, and apply Blaschke transform to x(t) to obtain the spectrum s, as shown in Eq (5).


s=BT(x) (5)

Perform mean truncation on the spectrum s and use a maximum value filter to extract the main trend of the spectrum, denoted as z, as shown in Eq (6).


$$z\left(n\right)=\underset{k\in [n-\frac{M-1}{\text{2}},n+\frac{M-1}{\text{2}}]}{max}\left(\left\{ \begin{array}{c} @l@s\left(k\right),\text{ if}s\left(k\right)\geq \mu \\ 0,\text{ if}s\left(k\right)<\mu \end{array}\right.\right)$$ (6)

where, M is the window size of maximum value filter, μ is the mean of s.

We can regard the amplitude z(i) as the distribution of sample number z(i) in position i, and regard the whole spectrum trend as the distribution of the sample N in {1,...,L}, then:


$$N=\sum_{i=1}^{L}z\left(i\right)$$ (7)

For a discrete distribution within the interval [a,b], define Q(a,b) as the proportion of samples on the interval [a,b], then:


$$Q\left(a,b\right)=\frac{\text{1}}{N}\sum_{i=a}^{b}z\left(i\right)$$ (8)

Assuming there is a discrete probability distribution rule $P=(p_{i}{)}_{i=1...L}$ applicable to spectral trend sequences z, we can regard P(a,b) as the possibility of sample points falling into the interval [a,b], then:


$$P\left(a,b\right)=\sum_{i=a}^{b}p_{i}$$ (9)

The hypothesis ${ℋ}_{\text{0}}$ holds that the spectrum band satisfies the discrete probability distribution law P. In other words, the distribution of N samples in the spectrum band F is independent.

In order to accept hypothesis ${ℋ}_{\text{0}}$, the best way is to test the similarity of Q(a,b) and P(a,b). Under the premise of the hypothesis ${ℋ}_{\text{0}}$, the probability that the interval [a,b] contains at least NQ(a,b) sample number can be given by the binary test hypothesis ℬ(N,NQ(a,b),P(a,b)), meanwhile, the probability that the interval [a,b] contains sample number less than NQ(a,b) is ℬ(N,N(1−Q(a,b)),(1−P(a,b))), where,


$$ℬ\left(n,k,p\right)=\sum_{j=k}^{n}\left(\begin{array}{c} @c@n\\ j \end{array}\right)p^{j}(1-p{)}^{n-j}$$ (10)

If these two probabilities are too small, that is, the hypothesis ${ℋ}_{\text{0}}$ is not valid, we define a false alarm probability:


$${NFA}_{p}\left(\left[a,b\right]\right)=\left\{ \begin{array}{c} @l@\frac{L(L+1)}{\text{2}}ℬ\left(N,NQ\left(a,b\right),P\left(a,b\right)\right)\text{ if}Q\left(a,b\right)\geq P\left(a,b\right)\\ \frac{L(L+1)}{\text{2}}ℬ\left(N,N\left(1-Q\left(a,b\right)),1-P(a,b\right)\right)\text{ if}Q\left(a,b\right)<P\left(a,b\right) \end{array}\right.$$ (11)

According to literature [20], when ${NFA}_{p}\left(\left[a,b\right]\right)\leq \frac{\text{1}}{\text{2}}$, the interval [a,b] is said to be a meaningful rejection of hypothesis ${ℋ}_{\text{0}}$.

Assume that $\left[z_{\text{1}},z_{\text{2}}\right]$ is a segment of z, where $z_{\text{1}}$ statistically satisfies a non-increasing characteristic and $z_{\text{2}}$ statistically satisfies a non-decreasing characteristic. In this case, the interval $\left[z_{\text{1}},z_{\text{2}}\right]$ is the mono-component spectrum interval.

To determine whether the segments $z_{\text{1}}$ and $z_{\text{2}}$ satisfy the statistical non-increasing or non-decreasing characteristics, we introduce a non-parametric density estimation method. By constructing the Grenander estimator of the normalized spectral trend segment in the discrete probability distribution space L, we obtain the closest approximation of a strictly monotonic non-increasing (or non-decreasing) sequence.

For a given trend segment $r=\left(r_{\text{1}},r_{\text{2}},\cdots ,r_{N}\right)$, define the D -transformation as shown in Eq (12).


$$\left\{ \begin{array}{c} @l@D(r{)}_{k}=\frac{r_{i}+\cdots +r_{\text{1}}}{j-i+1}\;k\in [i,j]\\ D(r{)}_{k}=r_{k}\text{ otherwise} \end{array}\right.$$ (12)

where, $r_{i}\leq r_{i+1}\leq \cdots \leq r_{j}$, and $r_{i-1}>r_{i}$, $r_{j+1}>r_{j}$.

We apply the D -transformation N times to r to obtain the closest approximation of a strictly monotonic non-increasing sequence (the non-decreasing sequence can be obtained using the symmetry principle), as shown in Eq (13).


$$r^{\left(N\right)}=D^{N}\left(r\right)$$ (13)

At this point, we can determine whether the segment has a statistical monotonic characteristic by measuring the similarity between the sequences r and $r^{\left(N\right)}$. Based on the ranges of the strictly monotonic non-increasing and non-decreasing sequences, we can then identify the segment as a mono-component spectrum.

The similarity measurement method is based on the Fine to Coarse algorithm [16], and its specific process is as follows.

Suppose 𝒫(L) is a discrete probability distribution space on {1,...,L}, where the discrete probability distribution vector $r_{t}=(r_{ti}{)}_{i=1,...L}$, and $\sum_{i=1}^{L}r_{ti}=1$. Define 𝒟(L)⊂𝒫(L) as the probability density space over {1,...,L}, then $r_{t}=(1/N)T\in 𝒫\left(L\right)$ is the observed normalized spectral segmentation.

Let ${\overset{―}{r}}_{t}$ be the unimodal symmetric estimation of $r_{t}$, and define the distribution of ${\overset{―}{r}}_{t}$ as the unique distribution on 𝒟(L) that satisfies the minimum KL divergence from $r_{t}$ to 𝒟(L). The expression is as follows:


$$KL\left(r_{t}\right|\left|{\overset{―}{r}}_{t}\right)=minKL\left(r_{t}\right|\left|p_{t}\right),\;p_{t}\in 𝒟\left(L\right)$$ (14)

where, $KL\left(r_{p}\right|\left|p_{t}\right)=\sum_{i=1}^{L}r_{ti}log(r_{ti}/p_{ti})$.

In the interval T={a,...,b}, let $P\left(a,b\right)={\overset{―}{r}}_{t}$, $Q\left(a,b\right)=r_{t}$, according to the hypothesis ${ℋ}_{\text{0}}$, the condition that T does not satisfy unimodal symmetry is:


$$NFA_{{\overset{―}{r}}_{t}}\left[a,b\right]\leq \delta$$ (15)

where δ is the set decision threshold.

When it is determined that the first half of a trend segment z satisfies the statistical monotonic increase and the second half satisfies the statistical monotonic decrease, this trend segment z can be recognized as a unimodal spectrum interval. Furthermore, the frequency spectrum range of the mono-component can be determined based on z range, and the mono-component can be obtained through inverse Fourier transform.

In summary, the steps of the proposed unimodal pre-segmentation strategy are as follows:

***Step 1:*** Perform Blaschke transform on the original signal to obtain the corresponding spectrum s.

***Step 2:*** Smooth the spectrum using mean truncation and maximum value filtering to obtain the spectral trend z.

***Step 3:*** Calculate the maxima and minima of the spectral trend, and segment z into a series of strictly monotonic intervals.

***Step 4:*** Set the decision threshold δ and use hypothesis testing to attempt merging all adjacent monotonic intervals into statistically monotonic intervals.

***Step 5:*** Merge the adjacent monotonic increasing and decreasing intervals to obtain the corresponding unimodal interval.

***Step 6:*** Based on the range of the unimodal interval, segment the original spectrum and perform inverse Blaschke transform to obtain the mono-component.

### 2.3. Group sparse filter for periodic pulse extracting

Periodic pulse characteristics are the most regular and interpretable components of a signal, revealing its underlying mechanisms. A key feature of periodic pulses is group sparsity [21], which allows for efficient extraction of intrinsic information. In this section, we design group sparsity filters to isolate the periodic pulses within the signal.

The energy of a periodic pulse signal’s frequency spectrum is typically concentrated in specific frequency bands, rather than isolated points, and exhibits a group sparse structure. Extracting the group sparse component of the frequency spectrum is crucial for signal decomposition. Group sparse constraints capture the continuous frequency band features of periodic pulses more accurately, avoiding misidentifications of isolated frequency points as in traditional periodic pulse models. Furthermore, while isolated frequency point sparsity is vulnerable to noise, group sparsity constraints enhance robustness by maintaining coherence across multiple frequency points within the band.

To accurately extract periodic pulse features and mitigate noise interference, EBMD introduces a novel filter: Group Sparse Filtering (GSF), designed to isolate the periodic pulse component of the signal. The key assumptions for the periodic pulse component are as follows: (1)The periodic pulse component in the frequency domain consists of distinct group sparse sub-components; (2) Each sub-component in the frequency domain has compact support, with elements within each support set being adjacent due to the mode’s bandwidth limitation; (3) The support sets of different sub-components are non-adjacent and have a defined interval.

Let $sig_{x}$ be the periodic pulse component, noise be the noise, and $sig_{x_{j}}$ be the group sparse sub-component. Then, the original signal y can be expressed as:


$$y=sig_{x}+noise=\sum_{j=1}^{N}sig_{x_{j}}+noise$$ (16)

Assume that there exists a specific group sparse filter $\hat{f}$, which, when $\hat{f}$ applied to the original signal y, can extract the periodic pulse features. This can be represented by the following equation:


$$sig_{x}=y\circ \hat{f}$$ (17)

where, ∘ is the Hadamard product and $\hat{f}$ is the ideal filter.

At this point, the corresponding group sparse filter can be obtained by solving the optimization formula shown in Eq (18).


$$\hat{f}=argmin𝒥\left(f\right):=\overset{\text{2}}{\underset{\text{2}}{\|c_{\text{y}}-c_{\text{y}}\circ f\|}}+\lambda {\|f\|}_{0,w}$$ (18)

where, λ is the regularization parameter, $\hat{f}$ is the ideal filter.

Furthermore, the *i-th* element of the group sparse filter can be represented by the following optimization.


$${\hat{f}}_{i}=argmin𝒥(f):=({𝐜}_{\text{y}}\left[i\right]{)}^{\text{2}}\left(1-f\right)+\lambda w_{i}{𝕀}_{\left\{f\right\}}\text{,i = 1, ... ,T}$$ (19)

where, $w_{i}$ is the weight coefficient.

By solving the above optimization problem based on the energy detection principle [21], an overall optimal group sparse filter with a group sparse structure can be obtained $\hat{f}$.

### 2.4. Periodic pulse fusion strategy and EBMD

Periodic pulses exhibit group sparsity, but decomposing the signal with a group sparsity filter often results in intrinsic modal components with similar periodic pulse features. These features are the most regular and interpretable components, revealing the signal’s underlying mechanisms. To prevent over-decomposition, we propose a fusion strategy based on the frequency correlation of periodic pulses. This strategy groups the fundamental mode components, with the following steps:

***Step 1:*** Set fusion threshold to thr,and define fundamental mode components as C.

***Step 2:*** Select the *i-th* signal from C, denoted as $C_{i}$. Perform the Hilbert transform on $C_{i}$ to obtain the envelope spectrum $f_{C_{i}}$, and denote the interval where the spectrum $f_{C_{i}}$ is greater than the mean as $L_{f_{C_{i}}}$. Let the maximum value of the periodic pulse frequency be ${\tilde{f}}_{C_{i}}$.

***Step 3:*** Select the *j-th* signal $C_{j}$ from C, and similarly calculate $f_{C_{j}}$, the envelope spectrum ${\tilde{f}}_{C_{j}}$, and the interval $L_{f_{C_{j}}}$.

***Step 4:*** Calculate the ratio of the overlapping length to the merging length of $L_{f_{C_{i}}}$ and $L_{f_{C_{j}}}$, denoted as r.

***Step 5:*** If r is greater than the set fusion threshold thr, and ${\tilde{f}}_{C_{j}}$ equal to ${\tilde{f}}_{C_{i}}$, then merge $C_{i}$ and $C_{j}$ to form a new $C_{i}$, and remove $C_{j}$ from the modal component matrix. Otherwise, no further action will be taken in this Step.

***Step 6:*** Return to ***Step 3*** and continue iterating through all the modal components in C until no further fusion with $C_{i}$ can occur. At this point, $C_{i}$ is considered the i-th intrinsic modal component $IMF_{i}$ obtained from the decomposition.

***Step 7:*** Return to ***Step 1*** until all fundamental mode components fusion to IMFs, the decomposition is completed.

As a new signal decomposition method, EBMD can accurately extract the periodic pulse features hidden in the signal, remove internal noise interference, and achieve signal separation, making it of practical application value. In summury, the basic steps of the EBMD algorithm are as follows:

***Step 1:*** Perform the Blaschke transform of the original signal and solve the Blaschke spectrum trend using mean truncation and maximum value filtering.

***Step 2:*** Use statistical hypothesis testing to divide the Blaschke spectrum into continuous intervals that satisfy the unimodal characteristics. Pre-segment the spectrum corresponding to the unimodal intervals into mono-component.

***Step 3:*** Perform a Fourier transform to obtain the spectrum of the mono-component, and apply energy detection principles to solve for the group sparse filter corresponding to each mono-component.

***Step 4:*** Employ the group sparse filter to extract the periodic pluse features of mono-component.

***Step 5:*** Perform inverse Fourier transform on the periodic pluse feature components to obtain the fundamental components.

***Step 6:*** Perform periodic pulse fusion strategy to fuse the fundamental mode components to IMF.

**Fig 1:**
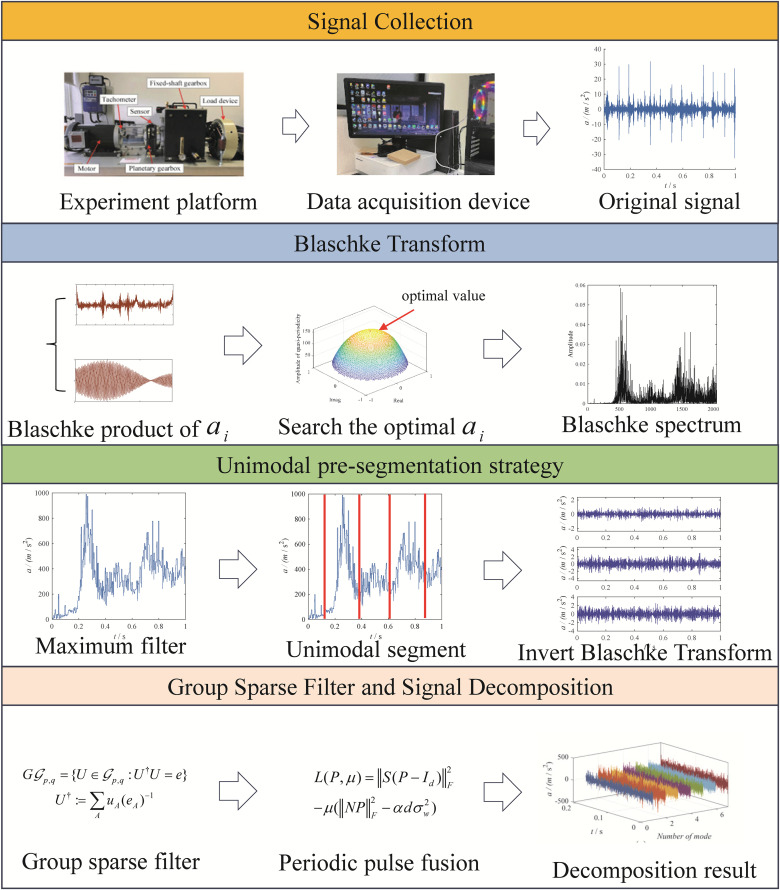
The flowchart of the EBMD.

The flowcharts of the EBMD signal decomposition is shown in Fig 1.

## 3. Experimental and analysis

As a novel signal decomposition method, EBMD is capable of decomposing signals into a series of periodic pulse feature components, significantly suppressing noise interference. This section demonstrates the superiority of EBMD through three experimental cases: fault feature extraction from gearbox vibration signals; EEG signal denoising; and ECG signal separation.

### 3.1. Case 1

Local faults in mechanical rotating components can generate periodic pulse transients in vibration signals, which manifest as group sparse characteristics in the frequency domain. However, these transients are often masked by strong background noise and fundamental vibration components (e.g., gear meshing frequencies, shaft rotation frequencies). Therefore, accurately extracting these periodic pulse transients is essential for mechanical fault diagnosis and maintenance.

**Fig 2:**
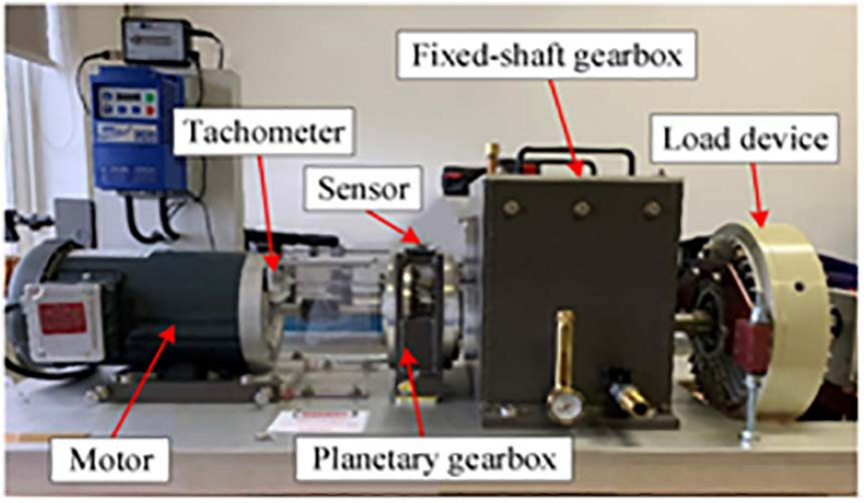
The planetary gearbox test bench configuration.

To validate the effectiveness of EBMD in extracting mechanical feature signals, we conducted experiments using the WT planetary gearbox dataset [22]. As shown in Fig 2, the experimental setup includes a motor, a planetary gearbox, a fixed-shaft gearbox, and a load device. Four planetary gears rotate around a central sun gear. Vibration data were collected with a Sinocera CA-YD-1181 accelerometer, and velocity pulses were recorded via an encoder. The sampling rate for all channels was set to 48 kHz.

**Fig 3:**
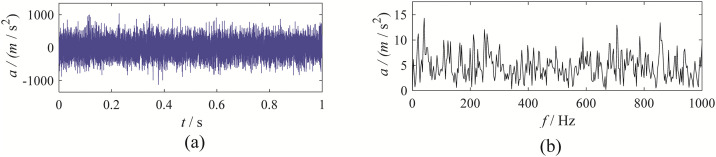
The time-domain waveform and envelope spectrum of gear fault signal: (a) time-domain waveform; (b) envelope spectrum.

This study utilizes gear tooth fault data to validate the performance of the EBMD method. The sun gear rotation frequency is set to 40 Hz, and the gear fault characteristic frequency is 125 Hz. To increase the decomposition difficulty, we added -5dB of Gaussian white noise to the original signal. Fig 3 displays the time-domain waveform of the gear fault and its envelope spectrum.

Upon observation, it can be seen that the gear vibration signal exhibits distinct amplitude and frequency modulation characteristics, which allow for a preliminary conclusion of a fault. However, its envelope spectrum is quite noisy, requiring further processing using signal decomposition methods.

We applied four decomposition methods—EBMD, VMD, EFD, and CEEMDAN—to process the gear signal. The parameters for each method are as follows:

- For EBMD, the pre-segmentation threshold was set to 0.5, and the peak filtering window was set to 100.
- For VMD, the number of modes was set to 8, with a penalty factor of 1800.
- For CEEMDAN, the added white noise level was 0.15dB, the mean frequency was set to 80, and the iteration count was 10.
- For EFD, the number of modes was set to 7.

**Fig 4:**
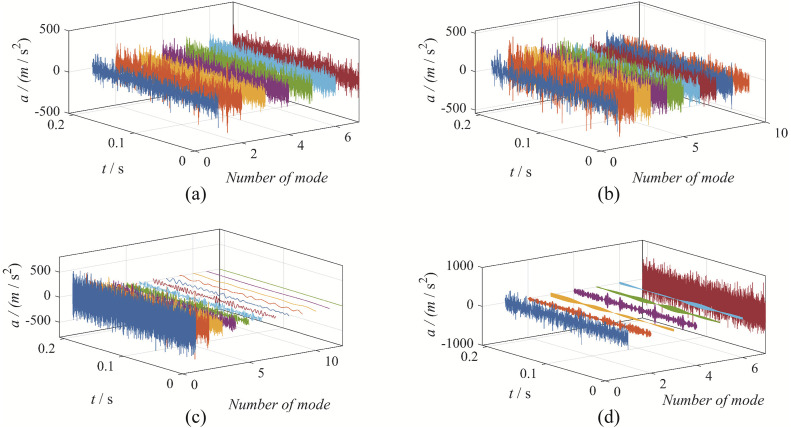
The decomposition result from the four decomposition methods: (a) EBMD; (b) VMD; (c) CEEMDAN; (d) EFD.

The time-domain waveforms obtained from the four decomposition methods are shown in Fig 4.

Upon observation, it is evident that EBMD decomposes the signal into 7 modes, VMD decomposes it into 8 modes, CEEMDAN decomposes it into 12 modes, and EFD decomposes it into 7 modes.

**Fig 5:**
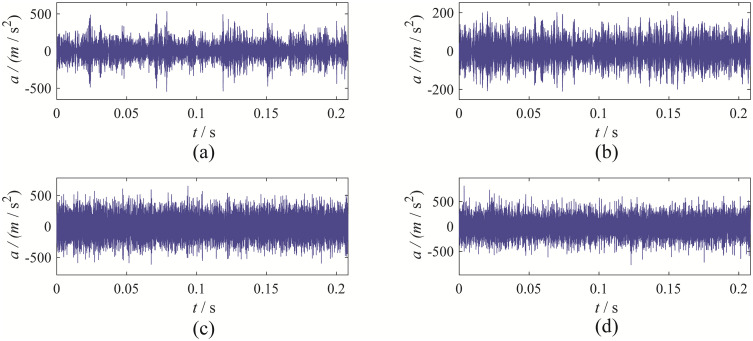
The time-domain of fault characteristic components from the four decomposition methods: (a) EBMD; (b) VMD; (c) CEEMDAN; (d) EFD.

Among these, the 2rd mode obtained by EBMD corresponds to the gear fault mode; the 2rd modes from VMD represent the gear fault characteristic components; the 1rd mode from CEEMDAN represents the gear fault characteristic component; the 7th modes from EFD represent the gear fault characteristic components, as shown in Fig 5.

**Fig 6:**
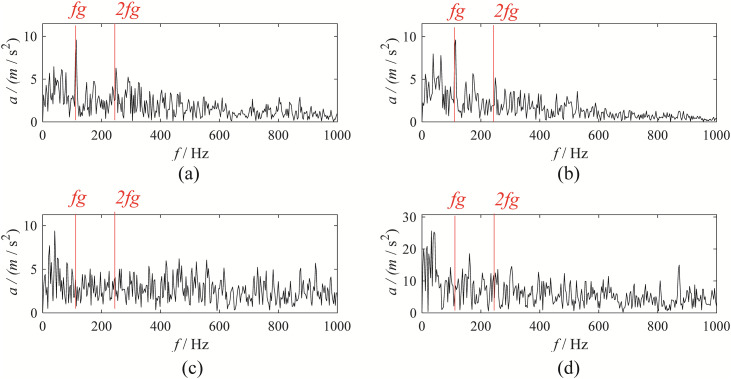
The evelope spectrum of fault characteristic components from the four decomposition methods: (a) EBMD; (b) VMD; (c) CEEMDAN; (d) EFD.

Compared to the original signal, the fault modes obtained by the four decomposition methods exhibit more distinct amplitude and frequency modulation characteristics. To further validate the effectiveness of the four methods, we performed envelope spectrum analysis on the fault modal components. The corresponding envelope spectra is shown in Fig 6.

Through observation, it can be seen that the envelope spectrum obtained by VMD exhibits some amplitude at the fault characteristic frequency and its harmonics, but the overall envelope spectrum is relatively noisy. The decomposition results of CEEMDAN and EFD are poor, with no obvious amplitude at the fault characteristic frequency, making it difficult to effectively assess the gear condition. In contrast, EBMD shows a clear amplitude at the fault characteristic frequency and its harmonics, with much less surrounding interference compared to EFD and VMD.

In conclusion, the signal decomposition ability of EBMD outperforms other algorithms, providing effective support for fault feature extraction from mechanical vibration signals.

### 3.2. Case 2

EEG signals, recorded through scalp electrodes, reflect the electrical activity of brain neurons. These signals are characterized by high temporal resolution and non-invasiveness, making them widely used in neuroscience research, clinical diagnosis (such as epilepsy detection), and brain-computer interfaces (BCI). However, EEG signals have low amplitude and poor signal-to-noise ratio, making them highly susceptible to contamination from environmental noise, such as power-line interference and electrode contact noise. Therefore, denoising is a critical step in EEG signal processing, directly affecting the reliability and effectiveness of subsequent analysis and applications.

**Fig 7:**
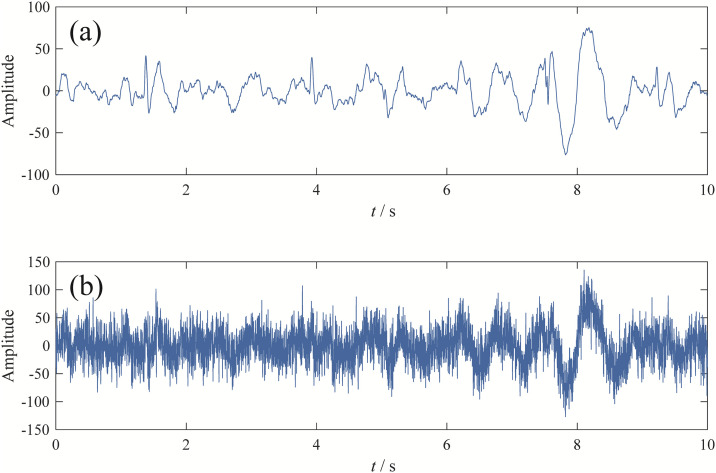
The time-domian waveform of EEG signal: (a) orignal signal; (b) signal with noise.

The dataset used in this study is from the Epileptic EEG Dataset [23], with a sampling frequency of 500 Hz and a signal length of 10 seconds. To verify the superiority of EBMD in EEG signal denoising, Gaussian white noise at -2dB was added to the original EEG signal. The time-domain waveforms of both the original and noisy EEG signals are shown in Fig 7.

Upon observation, it is evident that the noisy EEG signal has been completely contaminated, making it impossible to extract meaningful information. To address this, we employed four decomposition methods—EBMD, VMD, CEEMDAN, and EFD—to decompose the signal and extract the primary modal components. The parameters used for each method are as follows:

- For EBMD, the pre-segmentation threshold was set to 0.5, and the peak filtering window was set to 50.
- For VMD, the number of modes was set to 2, with a penalty factor of 1800.
- For CEEMDAN, the added white noise level was 0.15dB, the mean frequency was set to 80, and the iteration count was 10.
- For EFD, the number of modes was set to 2.

**Fig 8:**
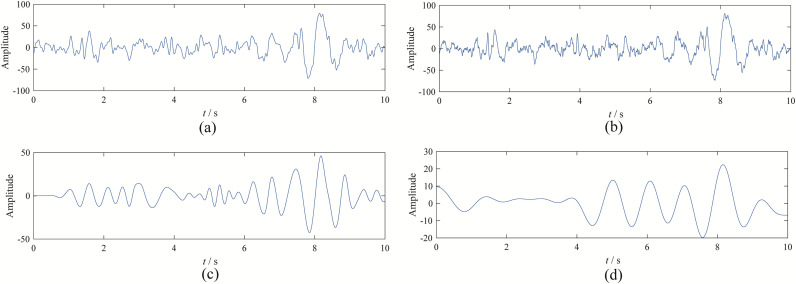
The decomposition result from four methods: (a) EBMD; (b) VMD; (c) CEEMDAN; (d) EFD.

We selected the component with the highest similarity to the original signal as the denoising result. The results obtained from the four decomposition methods are shown in Fig 8.

Upon observation, it can be seen that EBMD and VMD effectively denoise the signal, with the decomposed modes being very similar to the original signal. In contrast, EFD and CEEMDAN struggle to separate noise from the original signal, and the denoised results are distorted.

To further compare the decomposition performance of EBMD and VMD, we calculated the absolute error between the decomposition results of both methods and the original signal. The overall error for EBMD is $2.507\times 10^{\text{4}}$, while for VMD, it is $2.5664\times 10^{\text{4}}$. The absolute error plot also reveals that the signal obtained from EBMD is closer to the original signal compared to VMD.

In summary, compared to other methods, EBMD demonstrates superior signal decomposition performance, effectively removing signal noise and achieving denoising of the EEG signal.

### 3.3. Case 3

**Fig 9:**
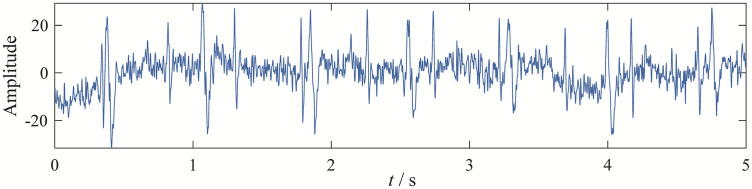
The time-domain waveform of original ECG signal.

Fetal electrocardiogram (ECG) signal analysis is of significant importance for monitoring fetal health. However, fetal ECG signals are often mixed with maternal ECG signals and other interference components, such as baseline drift. As shown in Fig 9, the original signal analyzed in this study is sourced from the Open Physiological Data website [24], with the original reference being. The sampling frequency of this signal is 1 kHz, and the sampling duration is 5 seconds.

To separate the fetal and maternal ECG signals, we applied four decomposition methods—EBMD, VMD, CEEMDAN, and EFD—to process the original signal. The parameters for each method are as follows:

- For EBMD, the pre-segmentation threshold was set to 0.5, and the peak filtering window was set to 100.
- For VMD, the number of modes was set to 6, with a penalty factor of 1800.
- For CEEMDAN, the added white noise level was 0.15dB, the mean frequency was set to 80, and the iteration count was 10.
- For EFD, the number of modes was set to 7.

**Fig 10:**
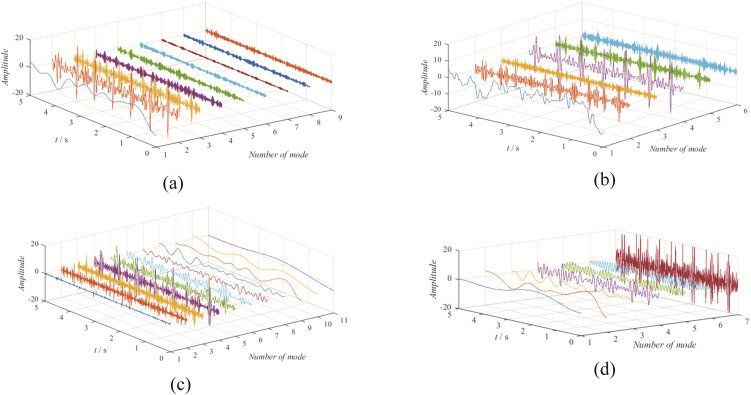
The decomposition results from four methods: (a) EBMD; (b) VMD; (c) CEEMDAN; (d) EFD.

The time-domain waveforms obtained from the four decomposition methods are shown in Fig 10.

**Fig 11:**
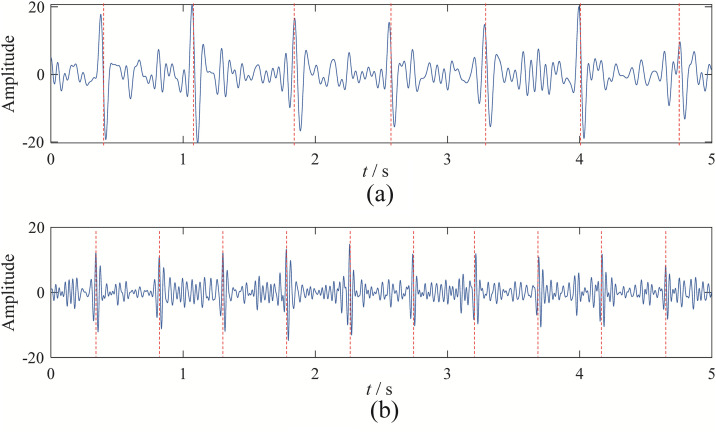
The fetal and maternal signal time domain wave of EBMD: (a) fetal signal; (b) maternal signal.

**Fig 12:**
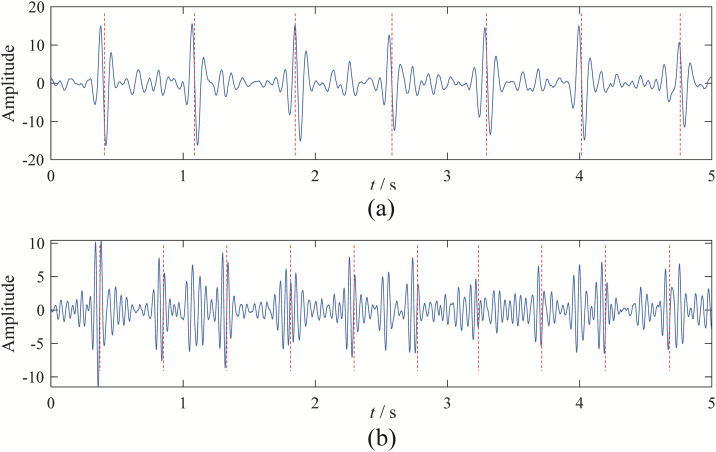
The fetal and maternal signal time domain wave of VMD: (a) maternal signal; (b) fetal signal.

**Fig 13:**
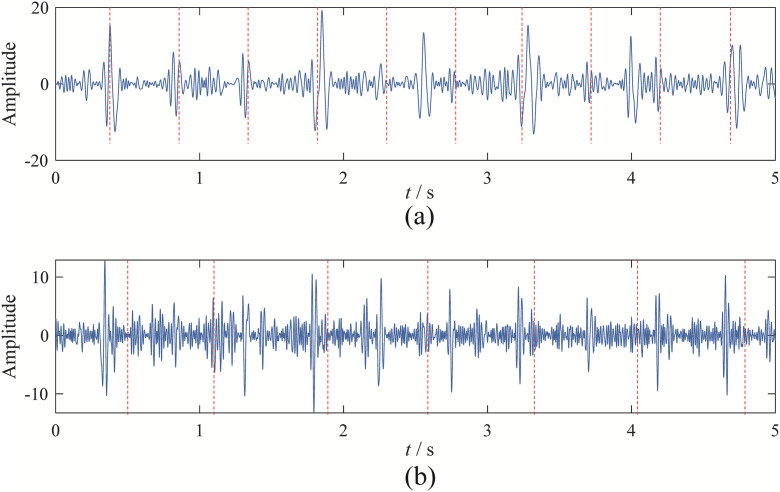
The fetal and maternal signal time domain wave of CEEMDAN: (a) maternal signal; (b) fetal signal.

**Fig 14:**
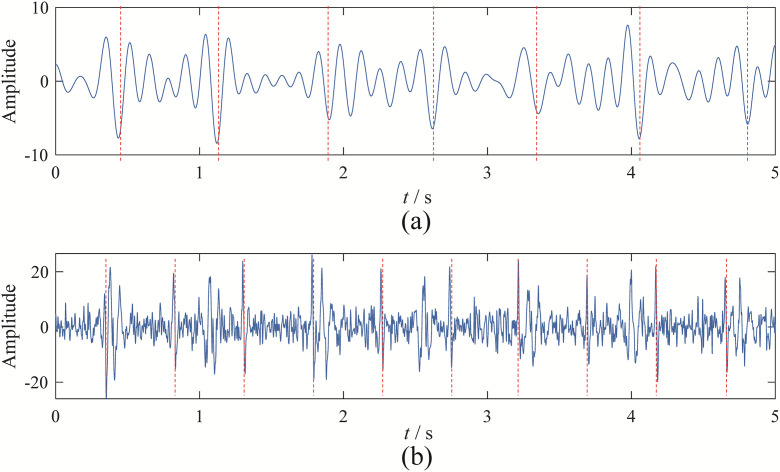
The fetal and maternal signal time domain wave of EFD: (a) maternal signal; (b) fetal signal.

Upon observation, it can be seen that EBMD decomposes the signal into 9 modes, VMD into 6 modes, CEEMDAN into 11 modes, and EFD into 7 modes. Among these, the 2nd and 3rd modes obtained from EBMD represent the fetal and maternal ECG components, as shown in Fig 11. The 4th and 5th modes from VMD correspond to the fetal and maternal ECG components, as shown in Fig 12. The 3rd and 4th components from CEEMDAN represent the fetal and maternal ECG components, as shown in Fig 13. Finally, the 4th and 7th components from EFD correspond to the fetal and maternal ECG components, as shown in Fig 14.

Upon observation, it is evident that EBMD successfully separates the target ECG signal and eliminates baseline drift. Although VMD can separate the maternal and fetal signals, the fetal ECG component still contains noise interference, making its overall performance inferior to EBMD. CEEMDAN can separate the maternal and fetal signals, but the separated components clearly contain significant noise. EFD, on the other hand, can only separate the fetal signal and struggles to eliminate the effects of baseline drift.

The main reason for these phenomena is that the maternal and fetal signals exhibit group sparsity characteristics in the frequency domain. VMD and EFD use Wiener and rectangular filters, respectively, which have difficulty accurately removing noise. CEEMDAN, by adaptively adding noise during the signal decomposition process, allows residual noise from each iteration to propagate to subsequent modes, resulting in poor decomposition performance.

It is worth noting that EBMD combines a single-modal pre-segmentation strategy and group sparse filtering approach, which allows it to accurately extract group sparse features and suppress noise interference. Therefore, EBMD outperforms other decomposition methods in ECG signal separation.

## 4. Conclusion

To address the core challenges of extracting periodic pulse features, this paper proposes a novel signal decomposition method, Empirical Blaschke Mode Decomposition (EBMD). First, EBMD employs a unimodal pre-segmentation strategy to define the Blaschke spectrum band range of the decomposition modes, ensuring their physical relevance. Next, EBMD utilizes a group sparse filter bank to decompose the signal into a series of fundamental modes, extracting the hidden periodic pulse features. Finally, EBMD introduces a periodic frequency similarity fusion strategy to consolidate the fundamental modes into eigenmode functions, effectively overcoming the over-decomposition issue. As a novel approach, EBMD enables effective mode separation and feature extraction, even in the presence of noise. The method is validated through a simulated case and three real-world applications: mechanical vibration signal feature extraction, EEG signal denoising, and ECG signal separation. Experimental results demonstrate that EBMD successfully separates signals, extracts features, and suppresses noise interference, highlighting its practical value. While EBMD shows strong performance in extracting periodic pulse features and reducing noise, its effectiveness is sensitive to parameter settings, particularly the maximum filter window value and the statistical threshold in the unimodal pre-segmentation strategy. These parameters significantly impact the decomposition results, and future research will focus on optimizing these parameters for better performance. Additionally, due to the high computational complexity of EBMD, particularly in the process of obtaining Blaschke products and applying group sparse filtering, future research will also explore the development of faster algorithms to reduce the processing time while maintaining decomposition accuracy.
